# Supplementary material for: Development and validation of a clinic machine-learning nomogram for the prediction of risk stratifications of prostate cancer based on functional subsets of peripheral lymphocyte
Source: J Transl Med. 2023 Jul 12;21:465. doi: 10.1186/s12967-023-04318-w (PMC10339548; doi:10.1186/s12967-023-04318-w)
Supplement: Supplementary file 1 — Additional file 1: Fig. S1. Boxplot of the quality control data of 41 characteristics in functional subsets of peripheral lymphocyte for 197 PCapatients enrolled in this study. Table S1. Performance evaluation of five ML algorithms in the training (first line in each cell) and test set (second line in each cell). Better results in the test set are shown in bold. Table S2. Comparison of this study with selected previous works. [file 12967_2023_4318_MOESM1_ESM.docx]

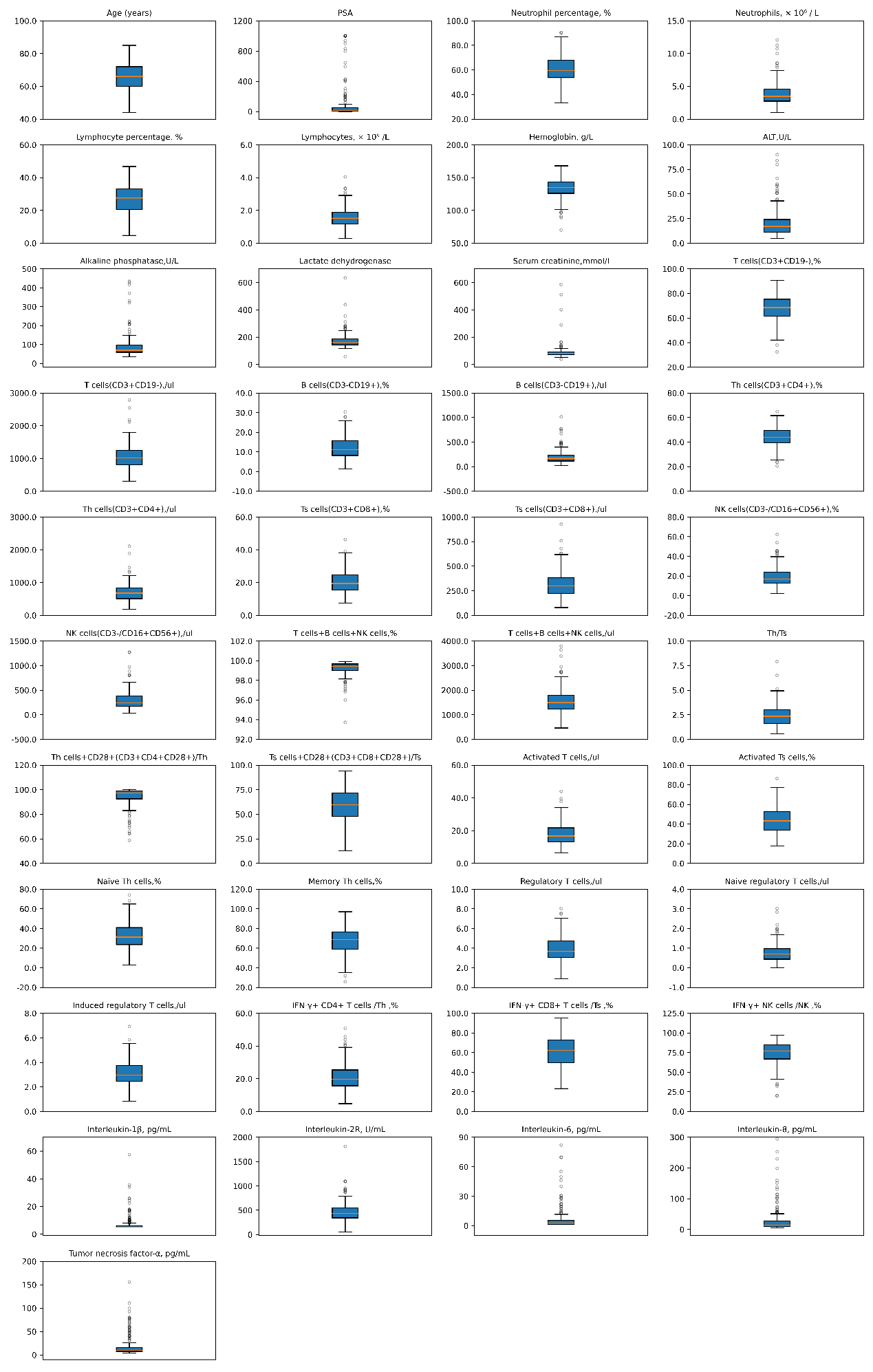


Fig. S1 Boxplot of the quality control data of 41 characteristics in functional subsets of peripheral lymphocyte for 197 PCa patients enrolled in this study

Table S1: Performance evaluation of five ML algorithms in the training (first line in each cell) and test set (second line in each cell). Better results in the test set are shown in bold.

| Models | Sensitivity  (95% CL) | Specificity  (95% CL) | F1  (95% CL) | AUC  (95% CL) |
| --- | --- | --- | --- | --- |
| AdaBoost | 0.840(0.780-0.897)  0.680(0.537-0.785) | 0.920(0.887-0.949)  0.837(0.757-0.917) | 0.841(0.791-0.884)  0.668(0.543-0.804) | 0.947(0.928-0.967)  0.761(0.663-0.859) |
| DTree | 0.975(0.946-0.994)  0.676(0.552-0.783) | 0.987(0.975-1.000)  0.838(0.753-0.911) | 0.974(0.955-0.990)  0.723(0.603-0.827) | 0.981(0.967-0.995)  0.756(0.672-0.840) |
| RF | 0.981(0.955-0.991)  **0.725(0.579-0.863)** | 0.984(0.970-0.998)  0.825(0.742-0.908) | 0.974(0.954-0.990)  **0.724(0.602-0.828)** | 0.998(0.995-1.000)  0.819(0.734-0.903) |
| SVM | 0.719(0.644-0.788)  0.624(0.500-0.738) | 0.860(0.823-0.899)  0.813(0.726-0.890) | 0.721(0.664-0.772)  0.620(0.492-0.739) | 0.867(0.832-0.901)  0.766(0.678-0.853) |
| XGBoost | 0.924(0.883-0.965)  0.680(0.535-0.825) | 0.963(0.933-0.993)  **0.853(0.743-0.963)** | 0.927(0.886-0.968)  0.664(0.518-0.810) | 0.989 (0.980-0.998)  **0.842(0.764-0.919)** |

Table S2: Comparison of this study with selected previous works

| Source | Classification Type | Number of patients | AUC |
| --- | --- | --- | --- |
| Osman et.al.,2019 [1] | low- *vs.* high-risk | 342 | 0.75 |
| Varghese et.al., 2019 [2] | low- *vs*. high-risk | 121 | 0.71 |
| Hood et.al., 2020 [3] | low-/intermediate-risk *vs.* high-risk | 126 | 0.853 |
| Montoya et.al., 2020 [4] | csPCa *vs*. non-csPCa | 80 | 0.92 (0.86-0.98) |
| Cosma et.al., 2021 [5] | low- *vs*. high-risk | 130 | 94.06% (±10.69) |
| Hiremath et.al., 2021 [6] | csPCa *vs*. non-csPCa | 592 | 0.81 (0.76–0.85) |
| Ours | low- *vs*. intermediate- *vs*. high-risk | 197 | 0.864 (0.794-0.935) |

**References for supplementary materials**

[1] Osman, S. O. S., Leijenaar, R. T. H., Cole, A. J., Lyons, C. A., Hounsell, A. R., Prise, K. M., O'Sullivan, J. M., Lambin, P., McGarry, C. K., & Jain, S. (2019). Computed Tomography-based Radiomics for Risk Stratification in Prostate Cancer. *International journal of radiation oncology, biology, physics*, 105(2), 448–456. https://doi.org/10.1016/j.ijrobp.2019.06.2504

[2] Varghese, B., Chen, F., Hwang, D., Palmer, S. L., De Castro Abreu, A. L., Ukimura, O., Aron, M., Aron, M., Gill, I., Duddalwar, V., & Pandey, G. (2019). Objective risk stratification of prostate cancer using machine learning and radiomics applied to multiparametric magnetic resonance images. *Scientific reports*, 9(1), 1570. https://doi.org/10.1038/s41598-018-38381-x

[3] Hood, S. P., Cosma, G., Foulds, G. A., Johnson, C., Reeder, S., McArdle, S. E., Khan, M. A., & Pockley, A. G. (2020). Identifying prostate cancer and its clinical risk in asymptomatic men using machine learning of high dimensional peripheral blood flow cytometric natural killer cell subset phenotyping data. *eLife*, 9, e50936. https://doi.org/10.7554/eLife.50936

[4] Montoya Perez, I., Jambor, I., Pahikkala, T., Airola, A., Merisaari, H., Saunavaara, J., Alinezhad, S., Väänänen, R. M., Tallgrén, T., Verho, J., Kiviniemi, A., Ettala, O., Knaapila, J., Syvänen, K. T., Kallajoki, M., Vainio, P., Aronen, H. J., Pettersson, K., Boström, P. J., & Taimen, P. (2020). Prostate Cancer Risk Stratification in Men With a Clinical Suspicion of Prostate Cancer Using a Unique Biparametric MRI and Expression of 11 Genes in Apparently Benign Tissue: Evaluation Using Machine-Learning Techniques. *Journal of magnetic resonance imaging*: JMRI, 51(5), 1540–1553. https://doi.org/10.1002/jmri.26945

[5] Cosma, G., McArdle, S. E., Foulds, G. A., Hood, S. P., Reeder, S., Johnson, C., Khan, M. A., & Pockley, A. G. (2021). Prostate Cancer: Early Detection and Assessing Clinical Risk Using Deep Machine Learning of High Dimensional Peripheral Blood Flow Cytometric Phenotyping Data. *Frontiers in immunology*, 12, 786828. https://doi.org/10.3389/fimmu.2021.786828

[6] Hiremath, A., Shiradkar, R., Fu, P., Mahran, A., Rastinehad, A. R., Tewari, A., Tirumani, S. H., Purysko, A., Ponsky, L., & Madabhushi, A. (2021). An integrated nomogram combining deep learning, Prostate Imaging-Reporting and Data System (PI-RADS) scoring, and clinical variables for identification of clinically significant prostate cancer on biparametric MRI: a retrospective multicentre study. *The Lancet. Digital health*, 3(7), e445–e454. https://doi.org/10.1016/S2589-7500(21)00082-0
